# Social cognition in children at familial high-risk of developing an eating disorder

**DOI:** 10.3389/fnbeh.2015.00208

**Published:** 2015-08-07

**Authors:** Radha Kothari, Manuela Barona, Janet Treasure, Nadia Micali

**Affiliations:** ^1^Behavioural and Brain Sciences, Institute of Child Health, University College LondonLondon, UK; ^2^Division of Psychology and Language Sciences, University College LondonLondon, UK; ^3^Psychological Medicine, King's College London Institute of PsychiatryLondon, UK; ^4^Department of Psychiatry, Icahn Medical School at Mount SinaiNew York, NY, USA; ^5^Mindich Child health and Development Institute, Icahn Medical School at Mount SinaiNew York, NY, USA

**Keywords:** ALSPAC, eating disorder, high-risk, social cognition, emotion recognition, SCDC, DANVA, phenotype

## Abstract

**Objective:** Diagnosis of an eating disorder (ED) has been associated with differences in social cognition. To date research investigating social cognition and ED has mainly employed patient and recovered samples. It is therefore unclear whether differences in social cognition are present prior to onset of ED, potentially contributing to development, or whether differences observed are a consequence of the disorder. We aimed to further explore whether individuals at high-risk for ED present social cognition characteristics previously found in ED groups.

**Methods:** Our sample was drawn from a population-based cohort, the Avon Longitudinal Study of Parents and Children (ALSPAC). Data on maternal ED behaviors over the lifetime were collected through in-depth clinical interviews (*n* = 1128) conducted using the Structured Clinical Interview for DSM disorders (SCID), and were used to categorize mothers according to ED behaviors over the lifetime: Restricting and Excessive Exercising (*n* = 58), Purging (*n* = 70), Binge-eating (*n* = 72), Binging and Purging (*n* = 66), no ED (*n* = 862). High-risk status of children was determined using these maternal lifetime behavioral phenotypes. Children at high-risk (maternal ED exposure) were compared to children at low-risk (born to mothers with no ED) on three measures of social cognition: the Social Communication Disorders Checklist (SCDC) (*n* = 922), the faces subtest of the Diagnostic Analysis of Non-Verbal Accuracy (DANVA) (*n* = 722), and the Emotional Triangles Task (*n* = 750).

**Results:** Children at high-risk for ED showed poorer performance on measures of social cognition compared to children at low-risk. Maternal lifetime binge-eating, and maternal lifetime binging and purging were associated with poorer social communication in children (OR: 2.4, 95% CI: 1.0, 5.7, *p* = 0.05; and OR: 2.7, 95% CI: 1.1, 6.5, *p* = 0.03 respectively). Maternal binging and purging was also found to be associated with differential facial emotion processing and poorer recognition of fear from social motion cues (B: −0.7, 95% CI: −1.1, −0.2, *p* = 0.004).

**Discussion:** Children at high-risk for ED showed slight differences in some areas of social cognition when compared to children at low-risk. Characteristic patterns in social cognition are present in children at high-risk for ED, particularly among children whose mothers have binge-eating and purging behaviors over the lifetime. Our findings support the hypothesis that these differences may be part of an intermediate phenotype for ED: perhaps contributing to development, or perhaps indexing a shared liability with psychiatric disorders characterized by abnormal social cognition.

## Introduction

Diagnosis of an eating disorder (ED) has been associated with difficulties in various aspects of socio-emotional processing: including emotion recognition, emotion regulation, interpersonal functioning, and theory of mind; both in the ill state and in recovery (Oldershaw et al., 2011a; Treasure et al., 2012; Dejong et al., 2013; Goddard and Treasure, 2013). Increasingly, research employing neuroimaging techniques has provided evidence for differences in socio-emotional processing among ED patients in areas of the brain that are also associated with eating and food, providing evidence for neural correlates of the socio-emotional difficulties observed among those with ED. Studies show that the neurocircuitry involved in the processing of food stimuli is altered among ED patients with regard to both reward and inhibition: specifically, ED patients appear to show an imbalance between the ventral neural system and the dorsal system (Friederich et al., 2013). The ventral system is important for identifying rewarding properties and attributing emotional significance to both food and non-food stimuli, leading to an emotional response. In contrast the dorsal system is important for emotion regulation and behavioral control (Favaro, 2013; Keel and Forney, 2013). The altered circuitry observed may contribute to both the deregulated eating and the deregulated social and emotional function characteristic of ED. ED also shows high comorbidity with other psychiatric disorders that have been associated with difficulties in social cognition. In comparison to the general population, increased rates of Autism Spectrum Disorders (ASD) and ASD type social difficulties have been observed among individuals with ED (Gillberg et al., 1994; Zucker et al., 2007; Oldershaw et al., 2011b), as have high levels of borderline personality disorder (BPD) (Sansone et al., 2004). ED and ED behaviors are also highly comorbid with social anxiety and social phobia (Halmi et al., 1991; Van Ameringen et al., 1991; Lépine and Pélissolo, 1996; Hinrichsen et al., 2003; Becker et al., 2004; Kaye et al., 2004; Swinbourne and Touyz, 2007), and it has been hypothesized that ED symptoms may in part be a method of coping with the altered emotional processing and social anxiety experienced. The extremely low weight characteristic of anorexia nervosa may also decrease feelings of anxiety by having a direct effect on serotonin levels and the HPA axis (Connan et al., 2003). Perhaps due to the wealth of evidence highlighting the importance of social cognition in ED, several theoretical models have implicated social and emotional difficulties as playing a key role in the development and maintenance of ED (Connan et al., 2003; Kaye, 2008; Hatch et al., 2010; Treasure et al., 2012).

The many studies showing that onset of anxiety disorders might pre-date onset of eating disorders have led to the hypothesis that early onset anxiety may increase vulnerability for an ED (Kaye et al., 2004; Swinbourne and Touyz, 2007; Micali et al., 2011). Though obsessive compulsive disorder (OCD) has often been considered to be the most highly comorbid anxiety disorder with ED, there is now much evidence showing that social anxiety and social phobia may also be highly comorbid (Powers et al., 1988; Halmi et al., 1991; Van Ameringen et al., 1991; Brewerton et al., 1993, 1995; Striegel Moore et al., 1993; Lépine and Pélissolo, 1996; Steiger et al., 1999; Godart et al., 2000; Hinrichsen et al., 2003; Swinbourne and Touyz, 2007), leading many to speculate that social anxiety might play a role in the development and etiology of ED (Brewerton et al., 1993; Becker et al., 2004). Taking into consideration evidence of a shared genetic liability for anxiety, depression, and ED symptoms (Silberg and Bulik, 2005), it is possible that difficulties in social cognition may predispose individuals to developing both social anxiety and ED, with the earlier onset of anxiety simply reflecting the natural course of both disorders. Alternatively, social anxiety may exist on the pathway between difficulties in social cognition and later ED (Kaye et al., 2004). It is important to note that for either of these hypotheses to be correct, difficulties in social cognition must be present prior to onset of ED.

### Are social cognition characteristics of ED premorbid?

It is known that having a psychiatric disorder can have an effect on the brain. Studies investigating schizophrenia and major depression show alterations in brain structure that are present in those with the disorder, but differ from those that are unaffected but at high-risk, indicating that these differences are a consequence of the disorder rather than being present premorbidly. In the case of ED the effects of weight loss or malnutrition, particularly during adolescence when the brain undergoes a period of increased development, may cause permanent or long lasting differences in brain structure that might be observable in ED patients and recovered individuals.

One method of investigating whether cognitive differences are present prior to onset of a disorder is to investigate a group at high-risk for that disorder (Kothari et al., 2013b, 2014a,b), and there is now a great deal of evidence to suggest that the first-degree relatives of ED probands are a high-risk group. Heritability estimates for ED range between 50 and 80% (Bulik et al., 2006; Leor et al., 2006; Klump et al., 2009; Mazzeo et al., 2009a,b), and prevalence is higher among the first-degree relatives of probands than among healthy or psychiatric controls (Holland et al., 1988; Strober et al., 1990, 2000; Ben-Dor et al., 2002). A recent study investigating the first-degree relatives of ED probands found that the fathers of ED patients show slight differences in response to social stimuli in comparison to healthy controls (Goddard and Treasure, 2013). It has been suggested however that risk factor studies in the ED field need to be conducted with participants young enough that they are unlikely to have developed any eating concerns (Lee et al., 2007). Children of women with an ED have been shown to be at high-risk for increased disordered eating and ED (Patel et al., 2002; Field et al., 2008; Goodman et al., 2014; Steinhausen et al., 2015). Moreover, research from our group has shown that children of mothers with AN have higher odds of emotional disorders in childhood/early adolescence (Micali et al., 2014b) The studies presented in this paper are the first to use the high-risk method to investigate whether differences in social cognition are present prior to onset by investigating children at high-risk. The children of mothers with lifetime ED (high-risk) are compared to children of non-ED women (low-risk) on measures of social communication (the Social Communication Disorders Checklist; SCDC), facial emotion recognition (Diagnostic Analysis of Non-Verbal Accuracy; DANVA), and interpretation of emotion from social motion cues (the Emotional Triangles Task). If differences in social cognition are observed among children at risk, this could indicate the presence of premorbid difficulties that may (i) be an intermediate phenotype and (ii) contribute to development of an ED.

### The problem of using diagnostic categories in research

Current diagnostic groupings of ED may not be effective for research regarding nosology and etiology; or in the search for biomarkers. Concerns have been raised regarding the lack of empirical evidence for the use of ED criteria (Hebebrand et al., 2004). In addition, the phenotypic expression of BN is very similar to that of AN-BP; however, the combination of binge eating and weight control behaviors means that individuals suffering from BN are generally at a normal weight. It has been suggested that the two diagnoses may only be distinguished by an ability to suppress weight to less than 85% of what would be expected (Polivy and Herman, 2002). Two particular concerns regarding the use of ED diagnoses in research are: (i) the heterogeneity of patients within the various ED diagnoses; and (ii) the instability of specific ED diagnoses over time (i.e., cross over between AN/BN/sub-threshold diagnoses). A high percentage of patients migrate between ED types (Fairburn and Harrison, 2003; Milos et al., 2005; Eddy et al., 2008), raising questions about true differences across ED diagnoses and highlighting problems with the validity of the DSM classifications. Evidence suggests that while cross-over from AN-R to AN-BP or BN is common, cross-over in the other direction is rarer (Milos et al., 2005; Tenconi et al., 2006; Eddy et al., 2008); possibly indicating an evidence-based distinction between AN-R and other ED diagnoses.

The considerable overlap of features between the different ED diagnoses, and the frequency of cross-over between these diagnoses, led Fairburn and Bohn to propose a “transdiagnostic” approach to ED classification (Fairburn and Bohn, 2005). The authors suggest that a category of “mixed eating disorders” is established, encompassing AN, BN, and EDNOS. They argue the case that the similarities between these disorders are more important than the differences; and that this approach would highlight the differences between the common traits of ED and traits associated with other psychiatric disorders, emphasizing the peculiar nature of the disorder. Though this approach has clear advantages, it may prove problematic with regard to research. The heterogeneity of existing ED diagnoses is already proving to be problematic when investigating the etiology and nosology of the disorders, and one overall group of ED may only make this research more difficult. There have been several studies attempting to empirically define ED phenotypes using latent class analysis (Keel et al., 2004), and more recently, Mazzeo and colleagues highlighted the importance of classifying ED at the symptom level (Mazzeo et al., 2009a). In their twin study investigating specific BN symptoms they found that while vomiting was very strongly influenced by additive genetic factors, other symptoms such as over concern with weight and shape were less heritable.

Anderluh and colleagues have previously suggested that a solution to the instability of ED diagnosis could be to classify individuals according to lifetime ED symptoms (Anderluh et al., 2009). They investigated this possibility and found that the four most common lifetime diagnostic categories, based on retrospective reporting, were: (i) Restricting subtype (no binging or purging present); (ii) Purging subtype (vomiting or other purging behaviors present); (iii) Binge/Purge subtype (binging and purging behaviors present); and (iv) Binging subtype (binging present without purging). They also found that: a longer duration of underweight status; longer episodes of severe restriction; episodes of excessive exercising; and shorter durations of binging were associated with perfectionism and rigidity.

Grouping participants with an ED according to the ED behaviors/symptoms that they have experienced over the course of their life would: (i) deal with limitations inherent in using current ED classification due to the instability of diagnoses; and (ii) circumvent limitations associated with the heterogeneity of ED diagnostic categories. In addition, it has been repeatedly suggested that a quantitative trait approach to psychiatric illness may be more relevant than current diagnostic categorization, particularly with respect to ED research (Zucker et al., 2007; Treasure, 2013). The National Institute of Mental Health (NIMH) has recently adopted this view, announcing a strategic plan to re-classify pathology based on observable behavior and neurobiological measures for the purposes of research. It has been suggested in this proposal that the limited clinical impact of recent research regarding mental health is due to new findings only moderately mapping onto current diagnostic categories; and this is because current diagnostic criteria are based on subjective clinical observation and patient symptom reports, rather than objective phenomenological and evidence based differences (Insel et al., 2010). Following on from this theoretical perspective, it is possible that findings from research regarding the cognitive profile and brain structure of ED individuals do not perfectly map onto diagnostic groups due to the instable and subjective nature of diagnostic criteria and diagnoses; and this could also partly explain conflicting evidence in the literature.

In the current study we propose that one way of addressing the instability and heterogeneity of ED diagnosis is using observable phenotypic features of ED (i.e., ED behaviors), which may be more directly associated with differences in cognitive functioning than DSM or ICD diagnoses. High-risk status of children was therefore determined using maternal lifetime ED behavioral phenotype, determined according to presence of ED behaviors over the lifetime (i.e., restricting, binge-eating, purging, and excessive exercising). Based on existing evidence we hypothesized that children at high-risk for ED would show differential performance on measures of social cognition; however, due to the novel nature of the research specific predictions could not be made and an exploratory approach was adopted.

## Methods

### Participants

Participants were drawn from the Avon Longitudinal Study of Parents and Children (ALSPAC), a population-based cohort of women recruited during pregnancy (*n* = 14.541) with the children that they were pregnant with at the time (Boyd et al., 2012; Fraser et al., 2013). Women were eligible for recruitment if they lived in a predefined area of the UK previously known as Avon; and if their expected date of delivery was between 1st April 1991 and 31st December 1992. Data have been collected since, from the mothers and resulting children, using questionnaires, biological sampling, and behavioral assessments conducted during clinics at the ALSPAC premises.

### Maternal ED behavioral phenotype (predictor)

As part of a larger study (Micali et al., in preparation), mothers from the ALSPAC cohort were assessed using a two-phase design for prevalence estimation. Women were not eligible for recruitment (*n* = 5076; 34.9% of the original cohort) if they were no longer enrolled in the ALSPAC cohort in 2009, if they had previously stated that they were unwilling to complete questionnaires, if their contact details were no longer known, or if they were experiencing difficulties of any kind that meant contact was unadvised (i.e., family bereavement). A target population of 9465 women (65.1% of the original cohort) participated in phase 1 (screening phase) and a total of 5716 women (60.4% of the target population) completed and returned questionnaires. Women screened positive for lifetime ED behaviors (*n* = 934 women, 16% of responses) if there was evidence of (i) weight and shape concerns; (ii) binging; and/or (iii) compensatory behaviors, following the algorithm used by Stice and colleagues for diagnosis of an ED using the EDDS (Stice, 2000), full details in Micali et al. (in preparation). All women who screened positive and a random 12% of screen negative women (*n* = 698) were eligible to take part in phase 2.

Women were interviewed using the Structured Clinical Interview for DSM-IV disorders (First et al., 2005), and the Lifeline section of The Longitudinal Interval Follow-up Evaluation (Keller et al., 1987). From the 1632 women in the selected sub-sample, interviews were conducted with 1110 (68%). Interviews were also conducted with an additional 33 women who did not participate in phase one, but had reported having had an ED in a previous questionnaire completed as part of ALSPAC. In total, data were collected from 1143 women, of whom 1128 had enough data for categorization according to lifetime behavioral phenotype, enabling us to determine risk status of their children (i.e., eligible for inclusion).

Based on the literature a hierarchical model was used to determine lifetime phenotype based upon ED behaviors presenting over the lifetime (Anderluh et al., 2003). Binging and purging behaviors trumped restricting and excessive exercising, and women were categorized into one of five groups:

1. Restricting and/or Excessive Exercising (no purging/no binging) = women who had, at any time in their life, engaged in dietary restriction for the equivalent of at least 1 day a week for a period of at least 3 months; and/or had engaged in excessive exercise to lose weight at a frequency of at least once a week for a period of at least 3 months. Women in this group must additionally have reached a BMI ≤ 18.5 at least once over their lifetime, and must never have binged or purged at a frequency of at least once a week for a period of at least 3 months (*n* = 58, 5.1% of mothers eligible for inclusion).
2. Purging (no binging) = women who had, at any time in their life, engaged in purging behaviors (i.e., vomiting/abuse of laxatives, diuretics or slimming pills) at a frequency of at least once a week for a period of at least 3 months, but had never engaged in binging behaviors at or above this threshold. Women in this group could additionally have engaged in restriction and/or excessive exercise to lose weight (*n* = 70, 6.2% of mothers eligible for inclusion).
3. Binge-eating (no purging) = women who had, at any time in their life, engaged in binge eating behaviors (with loss of control) at a frequency of at least once a week for a period of at least 3 months, but had never engaged in purging behaviors at or above this threshold. Women in this group could additionally have engaged in restriction and/or excessive exercise to lose weight (*n* = 72, 6.4% of mothers eligible for inclusion).
4. Binge-eating and Purging = women who had, at any time in their life, engaged in both binging and purging behaviors (not necessarily simultaneously) at a frequency of at least once a week for a period of at least 3 months. Women in this group could additionally have engaged in restriction and/or excessive exercise to lose weight (*n* = 66, 5.9% of mother eligible for inclusion).
5. Unexposed group = women who did not meet any of the above criteria were used as a comparison group (*n* = 862; 76.4% of mothers eligible for inclusion).

### Children's social cognitive function (outcome)

#### Social communication: social communication disorders checklist (SCDC)

The SCDC is a 12 item questionnaire, designed for parental completion, which measures social reciprocity and other verbal and non-verbal social traits characteristic of ASD. The SCDC has good internal consistency (0.93), high test—re-test reliability (0.81), and high heritability in both genders (0.74) (Skuse et al., 2005). A higher SCDC score is indicative of more difficulties in social communication, and the measure has been found to be predictive of ASD level traits with a sensitivity of 0.88 and a specificity of 0.91 when using a score of ≥ 9 out of 24 (Skuse et al., 2009). A detailed description of the measure has previously been published (Skuse et al., 2005, 2009). At 13.5 years of age, 7165 parents completed the SCDC for their children. Children were eligible for inclusion in analyses investigating social communication if data was available on this, maternal lifetime behavioral phenotype, and relevant confounding socio-demographic data (*n* = 922).

#### Facial emotion recognition: diagnostic analysis of non-verbal accuracy (DANVA)

The faces subtest of the DANVA (Nowicki and Duke, 1994) was used to assess facial emotion recognition of children in the ALSPAC cohort at 8.5 years of age (*n* = 7488). In this computerized task, participants are shown photographs of children's faces expressing happiness, sadness, anger, or fear, and are asked to identify which of the four emotions is being expressed. Higher scores on this task represent more errors or misattributions of emotions (lower accuracy). Binary scores, indicating whether children made more (above cut-off) or less (below cut-off) errors/misattributions, have been developed based on the distribution of results in the cohort. The cut-offs were determined by ALSPAC in collaboration with the creator of the task, Stephen Nowicki, and full details have previously been published (Kothari et al., 2013a). Children were eligible for inclusion in analyses investigating facial emotion recognition if data was available on this, maternal lifetime behavioral phenotype, and potentially confounding socio-demographic data (*n* = 722).

### Emotion recognition from social motion cues: emotional triangles task

This computer based assessment measures the participant's ability to attribute an emotional mental state to nonhuman animate entities. Participants are presented with a series of 5 s animations of a circle and a triangle moving around a screen. In 20 animations the shapes move around in a self-propelled and purposeful manner designed to evoke a mental state attribution of one of four emotions: happy, sad, angry, or scared. In another four animations the shapes move around the screen in a manner designed to look inanimate or “not alive.” Four outcome variables representative of accurate identification of each emotion are available, with higher scores being representative of better emotion recognition. More details on this measure have been previously published (Boraston et al., 2007; Kothari et al., 2013a). A total of 5844 children completed this task at 13.5 years of age, but children were only eligible for inclusion in analyses investigating emotion recognition from social motion cues if data was available on this, maternal lifetime behavioral phenotype, and potentially confounding socio-demographic data (*n* = 750).

### Socio-demographic data/covariates

Socio-demographic data on relationship status of mothers (married or cohabiting vs. not), maternal education (up to O level/GCSE equivalent vs. A level and above; A level and above equivalent in the United States to College board Advanced Placement Exams or SAT II), ethnicity (white vs. non-white), and parity (primiparous vs. multiparous), were collected during pregnancy via questionnaire. Maternal age data were collected at time of birth.

### Procedure

Ethical approval for this study was granted by the ALSPAC Law and Ethics Committee and Local Ethics Committees. Please note that the study website contains details of all the data that is available through a fully searchable data dictionary (http://www.bris.ac.uk/alspac/researchers/data-access/data-dictionary/).

### Data analysis

All variables were checked for inconsistencies/outliers using tabulations, graphs and plots. For participants with less than 25% missing data on the SCDC, total scores were calculated using prorating. The distribution of variables was inspected for normality; scores from the SCDC and the DANVA were not normally distributed and could not be transformed, therefore scores from these measures were used as binary variables according to pre-established cut-offs (described above). Differences in social cognition between children at high-risk (exposure to maternal lifetime ED) and those at low-risk (unexposed to maternal lifetime ED) were analyzed using linear regression (Emotional Triangles) and logistic regression (SCDC and DANVA). For inclusion into specific analyses data had to be available on maternal lifetime behavioral phenotype, potential confounders, and the relevant measure of social cognition (SCDC = 922; DANVA = 722; Emotional Triangles = 750; see Table 1 for breakdown across groups). Analysis of the overlap between the three samples showed that a total of 625 children were included in all three analyses (Breakdown across high-risk groups: Maternal Restricting/Excessive Exercising = 31, Maternal Purging = 30, Maternal Binge-eating = 36, and Maternal Binging and Purging = 33 children). Maternal lifetime behavioral phenotype was used to predict children's performance on each task, and analyses were conducted on each gender separately due to previous findings showing differences in performance between boys and girls (Kothari et al., 2013a). A priori confounders were included in a minimally adjusted model (model 1: child age, gender where appropriate, and tester). Potential confounders were investigated and additionally included in a second fully adjusted model, if associated with both predictor and outcome (model 2: maternal relationship status, age, and education; child parity and ethnicity). Socio-demographic predictors of attrition were included in all fully adjusted models. All analyses were conducted using SPSS version 21 and a two-tailed significance level of *p* ≤ 0.05 was used. Significance levels were not adjusted for multiple comparisons due to the exploratory nature of the study and the small differences expected in a high-risk study of this nature (Kothari et al., 2013b, 2014a,b).

**Table 1:** **Comparison of socio-demographic factors across phenotypic groups for each sample analyzed**.

	**No ED**	**Restricting/E.E**	**Purging**	**Binging**	**Binging and purging**
**SCDC**					
*n*	726	44	49	56	47
Gender: female (vs. male)	355 (48.9%)	15 (34.1%)	27 (55.1%)	35 (62.5%)	23 (48.9%)
Ethnicity: non-white (vs. white)	22 (3.0%)	2 (4.5%)	4 (8.2%)	2 (3.6%)	3 (6.4%)
Maternal education: A level and above (vs. up to O level)	365 (50.3%)	23 (52.3%)	29 (59.2%)	30 (53.6%)	26 (55.3%)
Parity: multiparous (vs. primiparous)	381 (52.5%)	21 (47.7%)	24 (49.0%)	26 (46.4%)	28 (59.6%)
Relationship status: married/cohabiting (vs. single/widowed/divorced)	611 (84.2%)	40 (90.9%)	39 (79.6%)	44 (78.6%)	37 (78.7%)
Age of mother at delivery	29.8 (4.4)	29.5 (4.2)	29.2 (5.4)	29.9 (5.0)	29.0 (4.3)
**DANVA**					
*n*	561	34	42	45	40
Gender: female (vs. male)	284 (50.6%)	12 (35.3%)	23 (54.8%)	26 (57.8%)	21 (52.5%)
Ethnicity: non-white (vs. white)	17 (3.0%)	1 (2.9%)	3 (7.1%)	2 (4.4%)	0 (0.0%)
Maternal education: A level and above (vs. up to O level)	288 (51.3%)	19 (55.9%)	23 (54.8%)	23 (51.1%)	25 (62.5%)
Parity: multiparous (vs. primiparous)	302 (53.8%)	16 (47.1%)	20 (47.6%)	21 (46.7%)	24 (57.1%)
Relationship Status: married/cohabiting (vs. single/widowed/divorced)	474 (84.5%)	33 (97.1%)	33 (78.6%)	37 (82.2%)	31 (77.5%)
Age of mother at delivery	30.0 (4.4)	29.6 (3.8)	29.6 (5.3)	29.4 (5.2)	28.8 (4.1)
**EMOTIONAL TRIANGLES**					
*n*	590	36	39	44	41
Gender: female (vs. male)	291 (49.3%)	12 (33.3%)	23 (59.0%)	30 (68.2%)	21 (51.2%)
Ethnicity: non-white (vs. white)	18 (3.1%)	2 (5.6%)	3 (7.7%)	1 (2.3%)	2 (4.9%)
Maternal education: A level and above (vs. up to O level)	299 (50.7%)	20 (55.6%)	23 (59.0%)	23 (52.3%)	24 (58.5%)
Parity: multiparous (vs. primiparous)	305 (51.7%)	18 (50.0%)	18 (46.2%)	19 (43.2%)	24 (58.5%)
Relationship status: married/cohabiting (vs. single/widowed/divorced)	504 (85.5%)	35 (97.2%)	31 (79.5%)	36 (81.8%)	31 (70.5%)
Age of mother at delivery	29.8 (4.4)	29.6 (3.8)	29.7 (5.3)	28.8 (4.9)	29.3 (4.2)

*1. Parity = presence of other children at time of pregnancy (multiparous) or not (primiparous)*.

*2. A level and above equivalent in the United States to College board Advanced Placement Exams or SAT II*.

*3. Mean (SD) presented for Age of Mother at Delivery; n (%) presented for all other socio-demographic variables*.

*4. SCDC = social communication disorders checklist; DANVA = diagnostic analysis of non-verbal accuracy*.

*5. Restricting/E.E. = restricting and/or excessive exercising*.

### Attrition

Logistic regression analyses were used to investigate predictors of attrition of maternal interview data (women not being interviewed), and attrition of children's data regarding social cognition (children not completing testing sessions). Older mothers (OR: 0.93, 95% CI:0.91, 0.95; *p* < 0.001) and mothers with higher educational qualifications (OR:0.63, 95% CI: 0.51, 0.79, *p* < 0.001) were more likely to have been interviewed, and therefore more likely to have been included in the current study. The children of older mothers and mothers with higher educational qualifications were also less likely to have missed testing sessions for the DANVA (OR: 0.91, 95% CI: 0.87, 0.94, *p* ≤ 0.001 and OR: 0.64, 95% CI: 0.46, 0.90, *p* = 0.01 respectively). Children who had (a) sibling(s) when they were born were more likely to have no data on the SCDC (OR: 1.40, 95% CI: 1.02, 1.92, *p* = 0.04). As described above, these variables were included in fully adjusted models (model 2).

## Results

### Socio-demographic characteristics

Socio-demographic characteristics for each sample analyzed (SCDC, DANVA, and Emotional Triangles) are presented in Table 1. The percentage of women that were married or cohabiting was particularly high among women with Lifetime Restricting/Excessive Exercising in comparison to all other groups, in all three sub-samples. It is also worth noting that the percentage of children of non-white ethnicity was low for all groups in all samples, as is the case for the ALSPAC cohort as a whole (Boyd et al., 2012; Fraser et al., 2013).

### Prevalence and correlates of lifetime ED behavioral phenotypes

#### Prevalence

Prevalence of lifetime ED behavioral phenotypes were calculated as a proportion of those women who completed phase one of the study, as is consistent with a two-phase design for prevalence estimation. Prevalence of women with a lifetime Restricting/Excessive Exercising phenotype was found to be lowest at 1.0%. Lifetime Purging and Lifetime Binging and Purging had a prevalence of 1.2%, while prevalence of Lifetime Binging was highest at 1.3%. Overall, a total of 4.7% of the sample met criteria for being in one of the lifetime ED phenotype categories (see Table 2).

**Table 2:** **Prevalence of eating disorder behaviors over the lifetime**.

	**Prevalence**
No ED	5450 (95.3%)
Lifetime restricting and/or excessive exercising	58 (1.0%)
Lifetime purging (with or without restricting and/or excessive exercising)	70 (1.2%)
Lifetime binge-eating (with or without restricting and/or excessive exercising)	72 (1.3%)
Lifetime binging and purging (with or without restricting and/or excessive exercising)	66 (1.2%)

*1. Weighted prevalence = weighted for whether mothers screened positive or negative in phase one of the two phase prevalence study*.

#### Social cognition

##### SCDC

Children of women with a Binging phenotype had higher odds of having poor social communication in the fully adjusted model (OR: 2.4, 95% CI: 1.0, 5.7, *p* = 0.05), and this was particularly the case for boys in both minimally (OR: 4.3, 95% CI: 1.5, 11.7, *p* = 0.01) and fully adjusted models (OR: 2.4, 95% CI: 2.2, 20.0, *p* = 0.001). Children of women with a Binging and Purging phenotype also had higher odds of having poor social communication in the fully adjusted model (OR: 2.7, 95% CI: 1.1, 6.5, *p* = 0.03). This was most marked in the daughters of women with a Binging and Purging phenotype in the fully adjusted model (OR: 3.4, 95% CI: 1.0, 11.3, *p* = 0.04). No other differences were observed (see Table 3).

**Table 3:** **Logistic regression analysis of children's social communication disorders checklist (SCDC) Scores: Comparison of high-risk and unexposed children (Odds ratios, 95% confidence intervals, and ***p***-values)**.

**SCDC**	**% Scoring > 8**	**Model 1 OR (95% C.I.)**	***p*-value**	**Model 2 OR (95% C.I.)**	***p*-value**
Unexposed	6.2	Ref.		Ref.	
R and EE	9.1	1.63 (0.61, 4.31)	0.33	1.38 (0.47, 4.09)	0.56
Purging	8.2	1.19 (0.41, 3.45)	0.75	1.49 (0.50, 4.44)	0.47
Binging	12.5	1.99 (0.85, 4.65)	0.11	2.40 (1.01, 5.74)	0.05
Binging and Purging	14.9	2.02 (0.87, 4.70)	0.10	2.72 (1.13, 6.54)	0.03
**GIRLS**					
Unexposed	5.9	Ref.		Ref.	
R and EE	6.7	0.91 (0.12, 7.15)	0.93	0.94 (0.12, 7.70)	0.96
Purging	3.7	0.47 (0.06, 3.62)	0.47	0.57 (0.07, 4.49)	0.60
Binging	2.9	0.47 (0.06, 3.58)	0.46	0.48 (0.06, 3.67)	0.48
Binging and Purging	17.4	2.84 (0.90, 8.94)	0.07	3.42 (1.04, 11.27)	0.04
**BOYS**					
Unexposed	6.5	Ref.		Ref.	
R and EE	10.3	2.12 (0.68, 6.58)	0.19	1.72 (0.47, 6.30)	0.41
Purging	13.6	2.22 (0.61, 8.05)	0.22	3.07 (0.81, 11.72)	0.10
Binging	28.6	4.26 (1.54, 11.73)	0.01	6.59 (2.18, 19.96)	0.001
Binging and Purging	12.5	1.52 (0.43, 5.36)	0.52	2.43 (0.66, 9.0)	0.18

*1. Odds of scoring above the established cut-off of ≥8 in comparison to the unexposed group*.

*2. R and EE = restricting and/or excessive exercising*.

*Model 1. Minimally adjusted model: adjusted for child age and gender, and interviewer*.

*Model 2. Fully adjusted model: adjusted for child age and gender, interviewer, child ethnicity, parity, maternal age at delivery, marital stability, and maternal education*.

##### DANVA

The children of women with a Binging and Purging phenotype had lower odds of making errors when recognizing emotion from high-intensity faces (OR: 0.27, 95% CI: 0.08, 0.93, *p* = 0.04), and lower odds of misattributing faces as sad (OR: 0.21, 95% CI: 0.05, 0.90, *p* = 0.04); differences remained significant in the fully adjusted model (see Table 4). No differences were observed when analyzing each gender separately (see Tables 5, 6).

**Table 4:** **Logistic regression analysis of children's facial emotion recognition scores: comparison of high-risk and unexposed groups (Odds ratios, 95% confidence intervals, and ***p***-values)**.

	**% Above cut-off**	**Model 1 OR (95% C.I.)**	***p*-value**	**Model 2 OR (95% C.I.)**	***p*-value**
**HAPPY FACES: ERRORS**					
Unexposed	23.4	–		–	
R and EE	20.6	0.61 (0.24, 1.51)	0.29	0.64 (0.26, 1.62)	0.35
Purging	21.4	0.89 (0.40, 2.00)	0.79	0.89 (0.40, 2.01)	0.89
Binging	31.1	1.49 (0.74, 3.02)	0.27	1.52 (0.75, 3.11)	0.25
Binging and purging	22.5	0.94 (0.42, 2.12)	0.88	0.96 (0.42, 2.19)	0.92
**SAD FACES: ERRORS**					
Unexposed	20.1	–		–	
R and EE	8.8	0.32 (0.09, 1.10)	0.07	0.33 (0.09, 1.15)	0.08
Purging	16.7	0.82 (0.34, 1.95)	0.65	0.82 (0.34, 1.97)	0.65
Binging	28.9	1.95 (0.95, 4.01)	0.07	1.96 (0.95, 4.06)	0.07
Binging and purging	7.5	0.03 (0.09, 1.03)	0.06	0.27 (0.08, 0.95)	0.04
**ANGRY FACES: ERRORS**					
Unexposed	15.9	–		–	
R and EE	11.8	0.55 (0.18, 1.72)	0.31	0.56 (0.18, 1.75)	0.32
Purging	21.4	1.24 (0.53, 2.86)	0.62	1.22 (0.52, 2.84)	0.64
Binging	15.6	1.01 (0.42, 2.46)	0.98	1.00 (0.41, 2.44)	1.00
Binging and purging	12.5	0.63 (0.23, 1.77)	0.38	0.60 (0.21, 1.74)_	0.35
**FEARFUL FACES: ERRORS**					
Unexposed	20.9	–		–	
R and EE	5.9	0.25 (0.06, 1.11)	0.07	0.25 (0.06, 1.12)	0.07
Purging	16.7	0.71 (0.30, 1.71)	0.45	0.72 (0.30, 1.73)	0.46
Binging	26.7	1.26 (0.61, 2.63)	0.53	1.26 (0.60, 2.62)	0.55
Binging and purging	15.0	0.69 (0.27, 1.76)	0.44	0.66 (0.26, 1.70)	0.39
**ALL FACES: ERRORS**					
Unexposed	26.2	–		–	
R and EE	11.8	0.33 (0.11, 1.02)	0.054	0.35 (0.12, 1.07)	0.07
Purging	21.4	0.73 (0.33, 1.62)	0.43	0.73 (0.32, 1.63)	0.44
Binging	28.9	1.12 (0.55, 2.26)	0.76	1.12 (0.55, 2.28)	0.76
Binging and purging	17.5	0.52 (0.22, 1.25)	0.14	0.47 (0.19, 1.15)	0.10
**LOW INTENSITY FACES: ERRORS**					
Unexposed	23.7	–		–	
R and EE	17.6	0.62 (0.24, 1.60)	0.32	0.64 (0.24, 1.65)	0.35
Purging	23.8	0.92 (0.43, 2.00)	0.84	0.95 (0.44, 2.06)	0.89
Binging	31.1	1.48 (0.74, 2.97)	0.27	1.49 (0.74, 3.01)	0.27
Binging and purging	25.0	1.01 (0.46, 2.22)	0.98	1.01 (0.45, 2.25)	0.98
**HIGH INTENSITY FACES: ERRORS**					
Unexposed	21.0	–		–	
R and EE	8.8	0.32 (0.09, 1.10)	0.07	0.33 (0.09, 1.16)	0.08
Purging	16.7	0.70 (0.29, 1.70)	0.43	0.70 (0.29, 1.70)	0.43
Binging	17.8	0.80 (0.35, 1.83)	0.60	0.80 (0.35, 1.85)	0.60
Binging and purging	7.5	0.27 (0.08, 0.93)	0.04	0.25 (0.07, 0.86)	0.03
**MISATTRIBUTED AS HAPPY**					
Unexposed	15.2	–		–	
R and EE	2.9	0.18 (0.02, 1.39)	0.10	0.19 (0.02, 1.44)	0.11
Purging	11.9	0.70 (0.25, 1.92)	0.48	0.74 (0.26, 2.05)	0.56
Binging	22.2	1.95 (0.87, 4.36)	0.11	2.00 (0.88, 4.51)	0.10
Binging and purging	12.5	0.74 (0.27, 2.04)	0.56	0.68 (0.25, 1.91)	0.47
**MISATTRIBUTED AS SAD**					
Unexposed	16.4	–		–	
R and EE	11.8	0.53 (0.17, 1.63)	0.27	0.54 (0.17, 1.68)	0.28
Purging	7.1	0.33 (0.10, 1.14)	0.08	0.31 (0.09, 1.08)	0.07
Binging	17.8	1.02 (0.44, 2.34)	0.97	1.00 (0.43, 2.31)	1.00
Binging and purging	5.0	0.21 (0.05, 0.90)	0.04	0.21 (0.05, 0.91)	0.04
**MISATTRIBUTED AS ANGRY**					
Unexposed	11.4	–		–	
R and EE	5.9	0.37 (0.08, 1.67)	0.20	0.35 (0.08, 1.63)	0.18
Purging	9.5	0.73 (0.24, 2.22)	0.58	0.71 (0.23, 2.19)	0.55
Binging	15.6	1.41 (0.57, 3.44)	0.46	1.38 (0.56, 3.39)	0.49
Binging and purging	10.0	0.81 (0.26, 2.51)	0.72	0.83 (0.27, 2.58)	0.74
**MISATTRIBUTED AS FEARFUL**					
Unexposed	20.1	–		–	
R and EE	17.6	0.71 (0.27, 1.83)	0.47	0.71 (0.27, 1.85)	0.48
Purging	21.4	1.12 (0.50, 2.51)	0.78	1.13 (0.50, 2.55)	0.77
Binging	28.9	1.78 (0.87, 3.63)	0.12	1.76 (0.86, 3.62)	0.12
Binging and purging	22.5	1.21 (0.53, 2.74)	0.65	1.16 (0.51, 2.65)	0.73

*1. Facial emotion recognition scores from Diagnostic Analysis of Non-verbal Accuracy (DANVA)*.

*2. R and EE = restricting and/or excessive exercising*.

*Model 1. Minimally adjusted model: adjusted for child age and gender, and tester*.

*Model 2. Fully adjusted model: adjusted for child age and gender, tester, interviewer, parity, ethnicity, maternal age at delivery, marital stability, and maternal education*.

**Table 5:** **Logistic regression analysis of female children's facial emotion recognition scores: comparison of high-risk and unexposed groups (Odds ratios, 95% confidence intervals, and ***p***-values)**.

	**% Above cut-off**	**Model 1 OR (95% C.I.)**	***p*-value**	**Model 2 OR (95% C.I.)**	***p*-value**
**HAPPY FACES: ERRORS**					
Unexposed	17.3	–		–	
R and EE	8.3	0.35 (0.04, 2.98)	0.34	0.33 (0.04, 2.88)	0.32
Purging	8.7	0.37 (0.08, 1.70)	0.20	0.35 (0.07, 1.68)	0.19
Binging	26.9	1.61 (0.59, 4.40)	0.35	1.89 (0.68, 5.29)	0.22
Binging and purging	14.3	0.95 (0.24, 3.72)	0.94	1.09 (0.27, 4.36)	0.91
**SAD FACES: ERRORS**					
Unexposed	18.7	–		–	
R and EE	16.7	0.93 (0.18, 4.86)	0.93	1.16 (0.22, 6.12)	0.87
Purging	17.4	1.09 (0.33, 3.63)	0.89	1.15 (0.34, 3.98)	0.82
Binging	23.1	1.75 (0.59, 5.20)	0.32	1.64 (0.55, 4.91)	0.38
Binging and purging	4.8	0.30 (0.04, 2.38)	0.25	0.29 (0.04, 2.39)	0.25
**ANGRY FACES: ERRORS**					
Unexposed	13.4	–		–	
R and EE	16.7	1.30 (0.24, 7.13)	0.77	1.30 (0.22, 7.72)	0.77
Purging	17.4	0.98 (0.29, 3.25)	0.97	0.77 (0.21, 2.75)	0.68
Binging	11.5	0.86 (0.22, 3.39)	0.83	0.75 (0.19, 3.06)	0.69
Binging and purging	14.3	0.94 (0.23, 3.89)	0.93	0.82 (0.19, 3.60)	0.79
**FEARFUL FACES: ERRORS**					
Unexposed	22.5	–		–	
R and EE	8.3	0.25 (0.03, 2.11)	0.20	0.21 (0.02, 1.86)	0.16
Purging	13.0	0.54 (0.18, 2.45)	0.54	0.61 (0.16, 2.31)	0.47
Binging	26.9	1.00 (0.38, 2.63)	0.99	0.95 (0.35, 2.53)	0.91
Binging and purging	19.0	1.21 (0.35, 4.16)	0.76	1.25 (0.36, 4.34)	0.73
**ALL FACES: ERRORS**					
Unexposed	26.8	–		–	
R and EE	16.7	0.56 (0.11, 2.84)	0.49	0.54 (0.10, 2.78)	0.46
Purging	17.4	0.60 (0.19, 1.93)	0.39	0.51 (0.16, 1.69)	0.27
Binging	26.9	0.93 (0.36, 2.44)	0.89	0.90 (0.34, 2.38)	0.83
Binging and purging	14.3	0.51 (0.14, 1.89)	0.31	0.48 (0.13, 1.79)	0.27
**LOW INTENSITY FACES: ERRORS**					
Unexposed	23.3	–		–	
R and EE	24.6	0.48 (0.09, 2.44)	0.37	0.45 (0.09, 2.37)	0.35
Purging	16.7	0.83 (0.28, 2.44)	0.74	0.79 (0.26, 2.36)	0.67
Binging	21.7	1.15 (0.45, 2.97)	0.77	1.13 (0.43, 2.92)	0.81
Binging and purging	30.8	1.05 (0.34, 3.31)	0.93	1.06 (0.33, 3.38)	0.93
**HIGH INTENSITY FACES: ERRORS**					
Unexposed	19.7	–		–	
R and EE	8.3	0.40 (0.04, 3.53)	0.41	0.33 (0.04, 3.09)	0.33
Purging	17.4	0.77 (0.23, 2.57)	0.66	0.56 (0.16, 2.03)	0.38
Binging	11.5	0.61 (0.16, 2.31)	0.47	0.58 (0.15, 2.26)	0.43
Binging and purging	0.0	0.00 (0.00, –)	1.00	1.00 (0.00, –)	1.00
**MISATTRIBUTED AS HAPPY**					
Unexposed	15.7	–		–	
R and EE	14.1	0.00 (0.00, –)	1.00	0.00 (0.00, –)	0.65
Purging	0.0	0.77 (0.15, 3.80)	0.74	0.68 (0.14, 3.48)	0.65
Binging	8.7	1.34 (0.42, 4.26)	0.62	1.33 (0.41, 4.24)	0.64
Binging and purging	19.2	1.43 (0.33, 6.16)	0.63	1.17 (0.26, 5.19)	0.84
**MISATTRIBUTED AS SAD**					
Unexposed	15.5	–		–	
R and EE	16.7	0.75 (0.14, 3.93)	0.73	0.79 (0.14, 4.58)	0.79
Purging	8.7	0.46 (0.10, 2.16)	0.32	0.43 (0.09, 2.15)	0.31
Binging	15.4	0.94 (0.28, 3.11)	0.92	1.06 (0.31, 3.64)	0.93
Binging and purging	4.8	0.19 (0.02, 1.62)	0.13	0.17 (0.02, 1.51)	0.11
**MISATTRIBUTED AS ANGRY**					
Unexposed	10.1	–		–	
R and EE	12.3	0.45 (0.05, 4.03)	0.48	0.33 (0.04, 3.14)	0.34
Purging	8.3	0.97 (0.25, 3.75)	0.97	0.73 (0.18, 3.05)	0.67
Binging	13.0	1.47 (0.43, 5.05)	0.54	1.35 (0.38, 4.76)	0.65
Binging and purging	15.4	0.80 (0.15, 4.13)	0.79	0.84 (0.15, 4.61)	0.84
**MISATTRIBUTED AS FEARFUL**					
Unexposed	21.1	–		–	
R and EE	16.7	0.65 (0.13, 3.37)	0.61	0.66 (0.12, 3.53)	0.63
Purging	17.4	0.85 (0.26, 2.78)	0.79	0.74 (0.22, 2.49)	0.63
Binging	30.8	1.88 (0.72, 4.93)	0.19	1.84 (0.69, 4.93)	0.23
Binging and purging	14.3	0.74 (0.20, 2.83)	0.66	0.62 (0.16, 2.46)	0.50

*1. Facial emotion recognition scores from Diagnostic Analysis of Non-verbal Accuracy (DANVA)*.

*2. R and EE = restricting and/or excessive exercising*.

*Model 1. Minimally adjusted model: adjusted for child age and gender, and tester*.

*Model 2. Fully adjusted model: adjusted for child age and gender, tester, interviewer, parity, ethnicity, maternal age at delivery, marital stability, and maternal education*.

**Table 6:** **Logistic regression analysis of male children's facial emotion recognition scores: comparison of high-risk and unexposed groups (Odds ratios, 95% confidence intervals, and ***p***-values)**.

	**% Above cut-off**	**Model 1 OR (95% C.I.)**	***p*-value**	**Model 2 OR (95% C.I.)**	***p*-value**
**HAPPY FACES: ERRORS**					
Unexposed	29.6	–		–	
R and EE	27.3	0.65 (0.21, 1.97)	0.44	0.64 (0.20, 2.00)	0.44
Purging	36.8	1.37 (0.46, 4.08)	0.57	1.45 (0.47, 4.46)	0.52
Binging	36.8	1.42 (0.49, 4.15)	0.52	1.34 (0.44, 4.11)	0.61
Binging and purging	31.6	0.80 (0.26, 2.43)	0.69	0.82 (0.27, 2.54)	0.73
**SAD FACES: ERRORS**					
Unexposed	21.7				
R and EE	4.5	0.14 (0.02, 1.18)	0.07	0.15 (0.02, 1.25)	0.08
Purging	15.8	0.80 (0.19, 3.32)	0.76	0.88 (0.21, 3.75)	0.86
Binging	36.8	3.01 (0.99, 9.16)	0.053	2.81 (0.88, 8.92)	0.08
Binging and purging	10.5	0.29 (0.06, 1.46)	0.13	0.26 (0.05, 1.36)	0.11
**ANGRY FACES: ERRORS**					
Unexposed	18.4				
R and EE	9.1	0.39 (0.07, 2.08)	0.27	0.40 (0.07, 2.15)	0.29
Purging	26.3	1.23 (0.33, 4.53)	0.76	1.48 (0.37, 6.00)	0.58
Binging	21.1	1.27 (0.36, 4.53)	0.71	1.36 (0.36, 5.22)	0.65
Binging and purging	10.5	0.36 (0.06, 2.15)	0.26	0.34 (0.05, 2.27)	0.26
**FEARFUL FACES: ERRORS**					
Unexposed	19.1				
R and EE	4.5	0.31 (0.04, 2.55)	0.28	0.31 (0.04, 2.56)	0.28
Purging	21.1	0.84 (0.23, 3.00)	0.79	0.99 (0.27, 3.67)	0.99
Binging	26.3	1.67 (0.49, 5.73)	0.41	1.58 (0.44, 5.63)	0.48
Binging and purging	10.5	0.36 (0.07, 1.82)	0.22	0.34 (0.07, 1.81)	0.21
**ALL FACES: ERRORS**					
Unexposed	25.6				
R and EE	9.1	0.24 (0.05, 1.27)	0.09	0.25 (0.05, 1.33)	0.10
Purging	26.3	0.76 (0.22, 2.60)	0.67	0.94 (0.27, 3.30)	0.92
Binging	31.6	1.48 (0.49, 4.52)	0.49	1.43 (0.43, 4.71)	0.56
Binging and purging	21.1	0.46 (0.13, 1.67)	0.24	0.45 (0.12, 1.73)	0.24
**LOW INTENSITY FACES: ERRORS**					
Unexposed	22.7				
R and EE	18.2	0.76 (0.22, 2.66)	0.67	0.85 (0.24, 3.03)	0.80
Purging	26.3	0.93 (0.28, 3.15)	0.91	1.16 (0.33, 4.09)	0.82
Binging	31.6	2.00 (0.64, 6.23)	0.23	1.83 (0.56, 5.93)	0.32
Binging and purging	26.3	0.95 (0.28, 3.21)	0.94	1.14 (0.33, 3.96)	0.84
**HIGH INTENSITY FACES: ERRORS**					
Unexposed	22.4				
R and EE	9.1	0.33 (0.07, 1.56)	0.16	0.35 (0.07, 1.67)	0.19
Purging	15.8	0.54 (0.14, 2.17)	0.39	0.59 (0.15, 2.43)	0.47
Binging	26.3	1.35 (0.43, 4.26)	0.61	1.34 (0.40, 4.51)	0.64
Binging and purging	15.8	0.53 (0.14, 2.05)	0.36	0.49 (0.12, 1.99)	0.32
**MISATTRIBUTED AS HAPPY**					
Unexposed	16.2				
R and EE	4.5	0.31 (0.04, 2.53)	0.28	0.31 (0.04, 2.53)	0.27
Purging	15.8	1.02 (0.25, 4.20)	0.98	1.06 (0.25, 4.52)	0.94
Binging	26.3	3.64 (0.98, 13.53)	0.054	3.68 (0.95, 14.27)	0.06
Binging and purging	10.5	0.46 (0.09, 2.25)	0.34	0.42 (0.08, 2.17)	0.30
**MISATTRIBUTED AS SAD**					
Unexposed	17.3				
R and EE	9.1	0.42 (0.08, 2.21)	0.30	0.40 (0.08, 2.16)	0.29
Purging	5.3	0.17 (0.02, 1.59)	0.12	0.15 (0.02, 1.44)	0.10
Binging	21.1	1.26 (0.36, 4.45)	0.72	1.19 (0.33, 4.32)	0.80
Binging and purging	5.3	0.16 (0.02, 1.51)	0.11	0.12 (0.01, 1.29)	0.08
**MISATTRIBUTED AS ANGRY**					
Unexposed	10.5				
R and EE	4.5	0.29 (0.03, 2.71)	0.28	0.29 (0.03, 2.85)	0.29
Purging	5.3	0.37 (0.04, 3.63)	0.39	0.42 (0.04, 4.40)	0.47
Binging	15.8	1.59 (0.36, 6.93)	0.54	1.35 (0.28, 6.50)	0.71
Binging and purging	10.5	0.78 (0.14, 4.37)	0.77	0.97 (0.16, 5.77)	0.98
**MISATTRIBUTED AS FEARFUL**					
Unexposed	19.1				
R and EE	18.2	0.80 (0.24, 2.71)	0.72	0.84 (0.25, 2.86)	0.78
Purging	26.3	1.46 (0.43, 4.97)	0.54	1.43 (0.41, 4.99)	0.58
Binging	26.3	1.96 (0.60, 6.42)	0.26	1.83 (0.55, 6.11)	0.33
Binging and purging	31.6	1.91 (0.61, 5.97)	0.27	1.83 (0.57, 5.89)	0.31

*1. Facial emotion recognition scores from Diagnostic Analysis of Non-verbal Accuracy (DANVA)*.

*2. R and EE = restricting and/or excessive exercising*.

*Model 1. Minimally adjusted model: adjusted for child age and gender, and tester*.

*Model 2. Fully adjusted model: adjusted for child age and gender, tester, interviewer, parity, ethnicity, maternal age at delivery, marital stability, and maternal education*.

##### Emotional triangles

Children of women with a Binging and Purging phenotype showed poorer recognition of fear (B: −0.7, 95% CI: −1.1, −0.2, *p* = 0.004), and this difference remained significant when adjusting for potential confounders (see Table 7). This was particularly the case for boys (Table 8). No other significant differences were observed (Table 9).

**Table 7:** **Linear regression analysis of children's emotion recognition from social motion cues: Comparison of high-risk and unexposed children (***B***-values, 95% confidence intervals, and ***p***-values)**.

		**m(SD)**	**Model 1 B (95% C.I.)**	***p*-value**	**Model 2 B (95% C.I.)**	***p*-value**	**R^2^**
Angry	Unexposed	2.60 (1.40)	Ref.		Ref.		
	R and EE	2.90 (1.68)	0.30 (−0.15,0.75)	0.19	0.44 (−0.02,0.90)	0.06	0.006
	Purging	2.70 (1.40)	0.11 (−0.33,0.55)	0.64	0.15 (−0.31,0.60)	0.53	0.001
	Binging	2.62 (1.36)	0.06 (−0.37,0.49)	0.77	0.04 (−0.40,0.47)	0.87	0.001
	Binging and purging	2.37 (1.69)	−0.23 (−0.66,0.20)	0.29	−0.19 (−0.62,0.25)	0.41	0.005
Happy	Unexposed	2.14 (1.60)	Ref.		Ref.		
	R and EE	1.93 (1.62)	−0.25 (−0.75,0.26)	0.34	−0.25 (−0.77,0.27)	0.35	0.005
	Purging	2.06 (1.62)	−0.04 (−0.54,0.45)	0.87	0.05 (−0.46,0.56)	0.86	0.004
	Binging	1.94 (1.66)	−0.21 (−0.69,0.27)	0.39	−0.25 (−0.75,0.24)	0.31	0.003
	Binging and purging	2.03 (1.69)	−0.08 (−0.56,0.40)	0.74	−0.16 (−0.65,0.34)	0.53	0.001
Sad	Unexposed	1.64 (1.25)	Ref.		Ref.		
	R and EE	1.46 (1.49)	−0.14 (−0.54,0.26)	0.49	−0.20 (−0.61,0.21)	0.35	0.005
	Purging	1.52 (1.21)	−0.16 (−0.55,0.23)	0.43	−0.21 (−0.61,0.19)	0.30	0.003
	Binging	1.51 (1.31)	−0.15 (−0.53,0.23)	0.43	−0.14 (−0.53,0.25)	0.48	0.003
	Binging and purging	1.46 (1.28)	−0.18 (−0.56,0.20)	0.35	−0.19 (−0.58,0.20)	0.33	0.000
Scared	Unexposed	2.16 (1.50)	Ref.		Ref.		
	R and EE	2.23 (1.49)	0.02 (−0.44,0.49)	0.93	0.10 (−0.38,0.58)	0.67	< 0.001
	Purging	2.20 (1.42)	0.07 (−0.39,0.52)	0.77	0.01 (−0.46,0.47)	0.98	< 0.001
	Binging	2.13 (1.26)	0.04 (−0.40,0.49)	0.85	0.07 (−0.38,0.53)	0.75	< 0.001
	Binging and purging	1.51 (1.73)	−0.65 (−1.09,-0.21)	0.004	−0.67 (−1.12,-0.21)	0.004	< 0.001

*1. R and EE = restricting and/or excessive exercising*.

*Model 1. Minimally adjusted model: adjusted for child age and gender, and tester*.

*Model 2. Fully adjusted model: adjusted for child age and gender, tester, ethnicity, parity, maternal age at delivery, maternal education, and marital stability*.

**Table 8:** **Linear regression analysis of male children's emotion recognition from social motion cues: Comparison of high-risk and unexposed children (***B***-values, 95% confidence intervals, and ***p***-values)**.

		***m* (SD)**	**Model 1 B (95% C.I.)**	***p*-values**	**Model 2 B (95% C.I.)**	***p*-values**	**R^2^**
Angry	Unexposed	2.72 (1.42)	Ref.		Ref.		
	R and EE	3.00 (1.83)	0.28 (−0.30, 0.86)	0.35	0.49 (−0.110, 1.08)	0.11	0.008
	Purging	2.94 (1.22)	0.25 (−0.44, 0.94)	0.48	0.27 (−0.42, 0.96)	0.45	0.006
	Binging	2.66 (1.58)	−0.07 (−0.80, 0.66)	0.86	−0.23 (−1.01, 0.55)	0.56	< 0.001
	Binging and purging	2.42 (1.43)	−0.31 (−0.91, 0.29)	0.31	−0.27 (−0.90, 0.36)	0.40	0.010
Happy	Unexposed	2.17 (1.69)	Ref.		Ref.		
	R and EE	1.62 (1.65)	−0.58 (−1.26, 0.1)	0.09	−0.66 (−1.36, 0.04)	0.06	0.026
	Purging	2.03 (1.44)	−0.18 (−0.99, 0.62)	0.65	−0.27 (−1.09, 0.55)	0.52	0.002
	Binging	1.97 (1.78)	−0.17 (−1.02, 0.69)	0.70	−0.21 (−1.13, 0.71)	0.65	0.004
	Binging and purging	1.75 (1.81)	−0.40 (−1.10, 0.30)	0.27	−0.55 (−1.30, 0.19)	0.14	0.014
Sad	Unexposed	1.54 (1.26)	Ref.		Ref.		
	R and EE	1.48 (1.57)	−0.07 (−0.57, 0.44)	0.79	−0.15 (−0.66, 0.36)	0.57	< 0.001
	Purging	1.44 (0.97)	−0.13 (−0.72, 0.47)	0.68	−0.11 (−0.71, 0.49)	0.73	0.002
	Binging	1.41 (0.97)	−0.12 (−0.75, 0.51)	0.70	−0.10 (−0.77, 0.57)	0.77	0.004
	Binging and purging	1.46 (1.01)	−0.07 (−0.59, 0.45)	0.79	0.14 (−0.68, 0.40)	0.61	0.002
Scared	Unexposed	2.37 (1.46)	Ref.		Ref.		
	R and EE	2.37 (1.43)	0.01 (−0.58, 0.61)	0.97	−0.003 (−0.62, 0.61)	0.99	0.000
	Purging	2.64 (1.30)	0.27 (−0.44, 0.97)	0.46	0.17 (−0.55, 0.89)	0.65	0.010
	Binging	2.25 (1.32)	−0.13 (−0.87, 0.61)	0.73	−0.03 (−0.84, 0.77)	0.94	0.002
	Binging and purging	1.58 (1.85)	−0.79 (−1.40,-0.17)	0.01	−0.80 (−1.45,-0.14)	0.02	0.053

*1. R and EE = restricting and/or excessive exercising*.

*Model 1. Minimally adjusted model: adjusted for child age and gender, and tester*.

*Model 2. Fully adjusted model: adjusted for child age and gender, tester, ethnicity, parity, maternal age at delivery, maternal education, and marital stability*.

**Table 9:** **Linear regression analysis of female children's emotion recognition from social motion cues: Comparison of high-risk and unexposed children (***B***-values, 95% confidence intervals, and ***p***-values)**.

		**m (SD)**	**Model 1 B (95% C.I.)**	***p*-value**	**Model 2 B (95% C.I.)**	***p*-value**	**R^2^**
Angry	Unexposed	2.47 (1.38)	Ref.		Ref.		
	R and EE	2.75 (1.44)	0.31 (-0.40, 1.03)	0.39	0.37 (−0.39, 1.13)	0.34	0.010
	Purging	2.54 (1.51)	0.08 (−0.49, 0.65)	0.78	0.16 (−0.44, 0.76)	0.74	< 0.001
	Binging	2.60 (1.27)	0.11 (−0.42, 0.63)	0.69	0.13 (−0.40, 0.66)	0.64	0.003
	Binging and purging	2.32 (1.98)	−0.11 (−0.73, 0.50)	0.71	−0.13 (−0.75, 0.50)	0.69	0.002
Happy	Unexposed	2.10 (1.50)	Ref.		Ref.		
	R and EE	2.44 (1.47)	0.35 (−0.43, 1.12)	0.38	0.57 (−0.24, 1.38)	0.17	0.010
	Purging	2.08 (1.76)	−0.02 (−0.64, 0.59)	0.94	0.17 (−0.47, 0.81)	0.60	< 0.001
	Binging	1.92 (1.63)	−0.19 (−0.76, 0.38)	0.51	−0.23 (−0.80, 0.34)	0.43	0.004
	Binging and purging	2.34 (1.52)	0.25 (−0.42, 0.91)	0.47	0.17 (−0.50, 0.84)	0.61	−0.006
Sad	Unexposed	1.73 (1.24)	Ref.		Ref.		
	R and EE	1.44 (1.39)	−0.28 (−0.93, 0.38)	0.41	−0.16 (−0.85, 0.53)	0.65	0.010
	Purging	1.58 (1.36)	−0.15 (−0.67, 0.37)	0.57	−0.23 (−0.78, 0.31)	0.40	0.004
	Binging	1.56 (1.47)	−0.18 (−0.66, 0.31)	0.47	0.16 (−0.64, 0.33)	0.52	0.004
	Binging and purging	1.45 (1.55)	−0.26 (−0.83, 0.30)	0.36	−0.26 (−0.83, 0.31)	0.38	0.010
Scared	Unexposed	1.94 (1.50)	Ref.		Ref.		
	R and EE	2.00 (1.60)	0.05 (−0.70, 0.80)	0.14	0.23 (−0.56, 1.02)	0.56	< 0.001
	Purging	1.90 (1.44)	−0.04 (−0.63, 0.56)	0.90	−0.11 (−0.73, 0.51)	0.73	< 0.001
	Binging	2.06 (1.24)	0.11 (−0.44, 0.66)	0.70	0.11 (−0.44, 0.66)	0.70	0.002
	Binging and purging	1.43 (1.62)	−0.50 (−1.15, 0.15)	0.13	−0.54 (−1.19, 0.11)	0.10	0.028

*1. R and EE = restricting and/or excessive exercising*.

*Model 1. Minimally adjusted model: adjusted for child age and gender, and tester*.

*Model 2. Fully adjusted model: adjusted for child age and gender, tester, ethnicity, parity, maternal age at delivery, maternal education, and marital stability*.

## Discussion

The aim of this study was to investigate whether specific differences in social cognition were present in children at high-risk for ED, in comparison to children at low risk. Due to limitations associated with using ED diagnoses in research, particularly when taking a lifetime approach to ED categorization (discussed above), a maternal lifetime behavioral phenotype was used to predict difficulties in social cognition among children at high-risk. Overall, findings showed slight differences among children of women with (i) lifetime Binging and (ii) lifetime Binging and Purging phenotypes, in comparison to children at low risk.

Difficulties in social communication were observed among children at high-risk, specifically among children exposed to maternal lifetime Binging and maternal lifetime Binging and Purging. Both girls and boys born to mothers with lifetime binging and purging showed poorer social communication than unexposed children. Our findings are in line with previous research showing social cognitive style characteristics as being specifically associated with binge-purge subtypes of ED vs. restrictive subtypes (Troop and Bifulco, 2002), The authors conclude that there are differences in etiology of ED subtypes, and that social cognitive styles may contribute to development of the binge-purge subtypes of ED. Our findings support these conclusions, and additionally suggest that difficulties in social communication may be particularly associated with risk for presenting with binging behaviors over the lifetime. Our findings are also in line with previous research showing that levels of social phobia are significantly higher among those with AN and BN in comparison to healthy controls, and are positively associated with levels of eating psychopathology among participants with BN particularly (Hinrichsen et al., 2003).

Our research showed no association between social communication difficulties and risk for ED of the non-binging type (i.e., maternal lifetime Restricting and Excessive Exercising or maternal lifetime Purging). It is possible to see this as being in contrast to previous findings which show an association between AN and difficulties in social and interpersonal function (Oldershaw et al., 2011a; Treasure et al., 2012); however it must be born in mind that the majority of studies investigating social communication and ED employ patients and recovered samples. Our findings could be an indication that deficits in social communication and interpersonal function observed in participants with AN are a result of low weight, or are a long-term scar of ED, and might therefore not be genetically-mediated or an intermediate phenotype. Alternatively these differences may be subtle amongst girls at high-risk and their phenotypic expression could be modulated by the presence of an ED. Our lifetime approach also reflects experience of ED over the lifetime, inclusive of diagnostic cross-over and the development of new symptoms. Given this, findings could be interpreted as showing an association between social communication difficulties and vulnerability to developing binge-eating. This is certainly a hypothesis that requires further investigation.

Children at high-risk due to maternal exposure to lifetime Binging and Purging showed comparatively poorer accuracy in recognition of fear from social motion cues (Emotional Triangles task). When analysing performance of each gender separately this difference only remained significant amongst boys; however a comparison of mean scores for each gender shows that while girls and boys at high-risk showed similar mean scores, the scores of boys and girls at low-risk were different. It is possible that this difference in fear recognition did not reach significance in girls due to smaller variance. Further research is required to determine whether this poorer fear recognition is specific to boys, or present in both genders at high risk for ED. Given the lower prevalence of ED among males compared to females, if poor fear recognition is specific to boys at high risk for ED this might suggest a gender specific effect and warrant further exploration.

Children at high-risk due to maternal exposure to lifetime Binging and Purging also showed slight differences in the recognition of faces, specifically they were less likely to make errors in the recognition of emotion from high-intensity faces (faces that expressed emotion intensely rather than subtly), and they were less likely to misattribute faces as sad. No differences were observed when analysing each gender separately. Our findings do not support previous research showing that patients with BN have difficulties when categorizing emotional faces (Zonnevijlle-Bendek et al., 2002; Kucharska-Pietura et al., 2004; Legenbauer et al., 2008; Pollatos et al., 2008; Kühnpast et al., 2012), but may indicate greater sensitivity to sad and intense facial expressions. Research into Event Related Potentials (ERPs) suggests individuals with BN process emotional faces differently in comparison to healthy controls, with increased cognitive effort being dedicated to the evaluation of facial expressions (Kühnpast et al., 2012). The authors suggest that this increased effort to identify emotions in others could lead to social interactions being more tiring for individuals with BN, which may motivate social withdrawal. Our findings do suggest that differential emotion recognition/processing is also associated with risk for ED, particularly with a binge-purge sub-type.

It has been suggested that deficits in the ability to categorize emotional information (i.e., faces) may contribute to interpersonal stress through misunderstandings in interpersonal relationships, and poor social communication on a day to day basis (Kühnpast et al., 2012). In addition, social anxiety has previously been associated with difficulties in identifying emotion from voices (McClure and Nowicki, 2001). Overall our findings indicate an association between both emotion recognition and social communication, and risk for ED of a binge-eating subtype over the lifetime. It is not possible to conclude from the current study whether the differences shown in emotion processing are contributing to the observed difficulties in social communication amongst children at high-risk for binging-type ED. Longitudinal research investigating this hypothesis might elucidate these findings.

Previous research conducted by our group has found evidence of high-prenatal testosterone exposure (Kothari et al., 2014a) in children at high-risk for BN, which is thought to be an intermediate phenotype for ASD and ASD type traits (i.e., poor emotion recognition and social communication) (Knickmeyer and Baron-Cohen, 2006; Auyeung et al., 2009). Previous research from our group has also shown a high prevalence of conduct disorder symptoms in boys at high risk for BN (Micali et al., 2014a). This supports the notion of social difficulties in boys at high-risk for BN. Evidence of such a relationship may lead to the development of interventions focused on improved emotion processing, which may in turn have a beneficial effect upon social functioning for individuals with binging-type ED.

### Strengths and limitations

This is the first study to investigate social cognition in children at high-risk for ED, and also the first to use lifetime behavioral phenotypes to determine risk status in children: the research has many strengths, but also limitations. Working with the ALSPAC has allowed the use of a large cohort of children. This is particularly beneficial with research investigating ED due to their low prevalence in the population. Because ALSPAC is a longitudinal study, data on cognitive function of children were collected prospectively. A limitation of this however is that the measures used to assess cognition could not be chosen based on evidence of their previous use in relation to ED. As a result, the measures used to assess social cognition of the children in this sample have had little or no use with ED populations, making comparison of the findings with existing literature more difficult. It is also worth noting that the SCDC was completed by mothers, and results may be a reflection of maternal deficits in social communication, interpersonal difficulties between the mother and child, or maternal reports being biased (shared method variance).

A strength of investigating cognition in a sample of children at risk (vs. a patient or recovered sample), is that findings are not a consequence of ED or attributable to the potential effects of limited nutritional intake. This is important as dietary restraint has been shown to have a negative effect on cognitive performance in children (Brunstrom et al., 2005), and adults (Keys et al., 1950; Green et al., 1994). The use of a non-clinical sample also means that it is possible to generalize the findings from these studies to subjects at high-risk for ED in the general population; however it is important to consider sample bias in relation to attrition. Women who participated in the assessment with their children were more likely to be older, more highly educated, and of a higher social class (Kothari et al., 2013b), which means that our findings are representative of a well educated population with a high socio-economic status. This type of limitation is characteristic of general population samples where participants are not selected for inclusion according to predefined criteria, and are outweighed by the increased power resulting from large samples and the generalizability of findings beyond clinical groups. Finally, findings must be considered in light of the fact that due to the exploratory nature of the study and the small differences expected, significance levels were not adjusted for multiple comparisons.

## Conclusion

Our findings show that children at high-risk for ED show differences in social cognition compared to those at low genetic risk; however, the exploratory nature of this study and the small differences observed mean that further research is required to determine the reliability of this finding. Differences observed may lead to difficulties with social interactions and increased social anxiety or problems in social interactions. In combination with neuropsychological difficulties previously observed in children at high-risk for ED (Kothari et al., 2013b), differences in social cognition may also be detrimental to one's problem solving ability, making it difficult for young people to navigate a social life which becomes increasingly complex during adolescence. Deficits in social communication and emotion perception may only become apparent and detrimental to functioning at this time, adding an additional obstacle to a period of intense internal and external change. Our findings support the notion of a possible shared liability for ED, externalizing disorders, social anxiety, and ASD. It is possible that maladaptive eating may result from social difficulties experienced at this time amongst high-risk children, or that other genetic, epigenetic and social influences may interact with an existing vulnerability to determine whether an individual develops an ED.

### Conflict of interest statement

The authors declare that the research was conducted in the absence of any commercial or financial relationships that could be construed as a potential conflict of interest.
